# In vivo Ebola virus infection leads to a strong innate response in circulating immune cells

**DOI:** 10.1186/s12864-016-3060-0

**Published:** 2016-09-05

**Authors:** Ignacio S. Caballero, Anna N. Honko, Stephen K. Gire, Sarah M. Winnicki, Marta Melé, Chiara Gerhardinger, Aaron E. Lin, John L. Rinn, Pardis C. Sabeti, Lisa E. Hensley, John H. Connor

**Affiliations:** 1 Bioinformatics Graduate Program, Boston University, Boston, MA USA; 2 Virology Division, United States Army Medical Research Institute of Infectious Diseases, Fort Detrick, MD USA; 3 Integrated Research Facility, National Institute of Allergy and Infectious Diseases, National Institutes of Health, Fort Detrick, MD USA; 4 Center for Systems Biology, Department of Organismic and Evolutionary Biology, Harvard University, Cambridge, MA USA; 5 Broad Institute of MIT and Harvard, Cambridge, MA USA; 6 Department of Stem Cell and Regenerative Biology, Harvard University, Cambridge, MA USA; 7 Department of Microbiology, Boston University School of Medicine, Boston, MA USA

**Keywords:** Ebola virus, Transcriptional response, Transcriptomics, Interferon-stimulated genes

## Abstract

**Background:**

Ebola virus is the causative agent of a severe syndrome in humans with a fatality rate that can approach 90 %. During infection, the host immune response is thought to become dysregulated, but the mechanisms through which this happens are not entirely understood. In this study, we analyze RNA sequencing data to determine the host response to Ebola virus infection in circulating immune cells.

**Results:**

Approximately half of the 100 genes with the strongest early increases in expression were interferon-stimulated genes, such as ISG15, OAS1, IFIT2, HERC5, MX1 and DHX58. Other highly upregulated genes included cytokines CXCL11, CCL7, IL2RA, IL2R1, IL15RA, and CSF2RB, which have not been previously reported to change during Ebola virus infection. Comparing this response in two different models of exposure (intramuscular and aerosol) revealed a similar signature of infection. The strong innate response in the aerosol model was seen not only in circulating cells, but also in primary and secondary target tissues. Conversely, the innate immune response of vaccinated macaques was almost non-existent. This suggests that the innate response is a major aspect of the cellular response to Ebola virus infection in multiple tissues.

**Conclusions:**

Ebola virus causes a severe infection in humans that is associated with high mortality. The host immune response to virus infection is thought to be an important aspect leading to severe pathology, but the components of this overactive response are not well characterized. Here, we analyzed how circulating immune cells respond to the virus and found that there is a strong innate response dependent on active virus replication. This finding is in stark contrast to in vitro evidence showing a suppression of innate immune signaling, and it suggests that the strong innate response we observe in infected animals may be an important contributor to pathogenesis.

**Electronic supplementary material:**

The online version of this article (doi:10.1186/s12864-016-3060-0) contains supplementary material, which is available to authorized users.

## Background

Ebola virus belongs to the family *Filoviridae* and is an envelope, non-segmented, negative-stranded RNA virus with filamentous virion morphology. Infection with Ebola virus causes Ebola Virus Disease, a disease associated with mortality rates between 25 and 90 % [1]. The earliest clinical symptoms are non-specific and flu-like, such as high fever, headache and myalgia [1–3]. During the large outbreak in West Africa (2014-present), the symptoms also included diarrhea and vomiting [2]. The late stage of disease is associated with immune cell imbalances such as neutrophilia and coagulation disorders like diffuse intravascular coagulopathy [4–6].

It has been postulated that survival following Ebola virus infection correlates with the ability of the host to mount an early and robust interferon response. In cultured human liver cells, the virus has been shown to block specific aspects of the innate immune response, preventing the expression of interferon-stimulated genes [7, 8]. Experiments in animal models, including mice [9, 10] and guinea pigs [11], have provided evidence that the suppression of interferon signaling is important for pathogenesis.

Previous studies of primates exposed to wild-type Ebola virus strains have suggested that there is a strong interferon-like transcription signal in circulating immune cells [12]. It has not yet been established whether interferon signaling is seen only in circulating immune cells or whether there is evidence for interferon-induced mRNA expression in infected tissues as well. It is also not well established whether this response is dependent on active virus replication. Several studies have reported that during infections caused by respiratory and hemorrhagic fever viruses, circulating immune cells undergo major gene regulatory changes that result in the upregulation of many innate immune system genes [13–16]. One of the most interesting aspects about this surge in transcriptional activity is that it constitutes an early measurable indicator of infection.

In the present study, we studied the transcriptional profile of circulating immune cells obtained from several different studies. We investigated the most robustly expressed genes that were observed in the peripheral blood mononuclear cells (PBMCs) of cynomolgus macaques following different routes of infection (aerosol and intramuscular), as well as following vaccination of the host. We further investigated whether genes highly-upregulated in PBMCs also showed changes in expression in tissues of infected animals. Our results suggest that interferon-signaling is an early and robust response to Ebola virus infection throughout the body.

## Methods

### Datasets

#### Macaque model of intramuscular exposure to Ebola virus

This RNA sequencing study used cynomolgus macaques divided in two groups: vaccinated and Ebola-naïve [17]. The vaccinated group received an intramuscular injection of recombinant Vesicular Stomatitis Virus with the Ebola glycoprotein (rVSV/EBOV-GP) and a lethal dose of EBOV strain Kikwit several days later. The Ebola-naïve group was treated with a non-protective dose of rVSV/MARV-GP and received the same lethal dose of EBOV administered to the vaccinated group several days later. Blood samples were taken at 0, 4 and 7 days post-infection (see Table 1). PBMCs were isolated from the 18 blood samples, and RNA sequencing was performed using the same methods specified in [18].

**Table 1:** Summarizes the datasets described below

Study	Ref.	Tissue	Group	Platform	Num. animals	Num. samples	Timepoints (samples/timepoint)
Macaque model of intramuscular exposure to Ebola virus	[17]	PBMC	rVSV/EBOV-GP (vaccinated)	RNAseq	9	9	0, 4, 7 dpi (*n* = 3)
			rVSV/MARV-GP (Ebola-naïve)	RNAseq	9	9	0, 4, 7 dpi (*n* = 3)
Macaque model of aerosol exposure to Ebola virus	[19]	PBMC	EBOV	RNAseq	8	12	0 (*n* = 4), 1 (*n* = 2), 3 (*n* = 2), 6 (*n* = 2), 8 (*n* = 2) dpi
				Microarray	14^a^	36^a^	0 (*n* = 18), 1 (*n* = 2), 3 (*n* = 2), 4 (*n* = 4), 5 (*n* = 4), 6 (*n* = 2), 7 (*n* = 2), 8 (*n* = 2) dpi
		Spleen		RNAseq	19	19	0 (*n* = 2), 1 (*n* = 3), 3 (*n* = 3), 4 (*n* = 2), 5 (*n* = 2), 6 (*n* = 2), 7 (*n* = 3), 8 (*n* = 2) dpi
		Adrenal gland			12	12	1 (*n* = 2), 3 (*n* = 1), 4 (*n* = 2), 6 (*n* = 1), 7 (*n* = 3), 8 (*n* = 2) dpi
		Lymph node			6	6	0, 1, 3, 4, 5, 6 dpi (*n* = 1)
		Brain			10	10	1, 3, 4, 6, 7 (*n* = 2)
		Liver			4	4	0, 3, 5, 8 dpi (*n* = 1)
		Pancreas			3	3	0, 4, 8 dpi (*n* = 1)
Mouse model of exposure to Ebola virus	[9]	Spleen	WT-EBOV	Microarray	15	15	0 (*n* = 2), 12 (*n* = 4), 24 (*n* = 3), 36 (*n* = 3), 48 (*n* = 3) hpi
			MA-EBOV		14	14	0 (*n* = 2), 12 (*n* = 3), 24 (*n* = 3), 36 (*n* = 3), 48 (*n* = 3) hpi
Macaque model of aerosol exposure to Lassa and Marburg virus	[24]	PBMC	LASV	RNAseq	7	12	0 (*n* = 4), 3 (*n* = 4), 6 (*n* = 2), 10 (*n* = 2) dpi
					9	12	0, 3, 5, 9 dpi (*n* = 3)

^a^ 8 of these animals (and 12 of these samples) correspond to the 8 animals (and 12 samples) that were quantified using RNA sequencing)

#### Macaque model of aerosol exposure to Ebola virus

This study used rhesus macaques exposed to EBOV via aerosol (between 7.43×10^2^ and 2.74×10^2^ plaque-forming units (PFU)) 12 PBMC samples were collected at 0, 3, 6 and 8 days post-infection (see Table 1 for details) [19]. 19 spleen samples were obtained at 0, 1, 3, 4, 5, 6, 7 and 8 dpi. 12 adrenal gland samples were obtained at 1, 3, 4, 6, 7 and 8 dpi. 8 axillary lymph node samples were obtained at 0, 1, 3, 4, 5 and 6 dpi. 11 brain samples were obtained at 1, 3, 4, 6 and 7 dpi. 4 liver samples were obtained at 0, 3, 5 and 8 dpi. 3 pancreas samples were obtained at 0, 4 and 8 dpi. RNA was extracted from PBMCs and the other tissues and sequenced [20, 21]. For tissue homogenates, RNA was extracted from TRIzol-inactivated samples using BCP:chloroform, and total RNA was depleted of small RNAs (<200 nt) with a mirVana miRNA Isolation Kit per manufacturer’s instructions (Thermo Fisher, Waltham MA). Subsequently, ribosomal RNA was depleted using complementary DNA oligos and RNase H. For PBMC samples, mRNA was enriched using the Dynabeads mRNA Purification Kit (Thermo Fisher) per protocol. For all samples, TruSeq v2 (Illumina, San Diego CA) libraries were constructed as described previously [20, 22], multiplexed using custom adaptors [20], and sequenced on a HiSeq to obtain 2 × 100 bp paired-end reads. Reads were demultiplexed with a 0 mismatch tolerance, and cross-sample contamination was back-calculated from synthetic RNAs that were spiked in prior to library construction [22]. The PBMC samples referenced above, as well as 24 additional PBMC samples taken at 0, 1, 3, 4, 5, 6, 7 and 8 dpi (Table 1), were also quantified using microarrays according to the methods specified in [23].

#### Mouse model of exposure to Ebola virus

This study used BALB/c mice divided into two groups [9]. One group was inoculated intraperitoneally with wild-type EBOV. The other group was inoculated with mouse-adapted EBOV. Spleen samples were collected and homogenized at 0, 12, 24, 48 and 72 h post-infection for both groups (see Table 1). RNA was extracted from the 29 spleen samples and Agilent microarray processing was performed as described in [9].

#### Macaque model of aerosol exposure to Lassa and Marburg virus

This study used cynomolgus macaques divided in two groups [24]. One group was exposed to LASV Josiah and the other to MARV Angola, both groups received 1000 PFU of virus via aerosol. RNA was extracted from 24 PBMC samples (see Table 1) and RNA sequencing was performed.

### Fold change analysis of sequencing data

After the raw sequencing reads of each study were generated, they were trimmed and the adapters were removed using Trimmomatic [25]. The processed reads were aligned to the Macaca mulatta genome (Ensembl release 77) [26] using TopHat 2.0.11 [27] with default parameters (segment length of 25, allowing up to 2 segment mismatches). Gene counts were obtained using HTSeq [28] to count reads that aligned uniquely to each gene. Counts were normalized to compensate for differences in library size using the trimmed mean of M-values normalization method included in the edgeR BioConductor package [29].

A gene was deemed to show statistically significant changes in expression at a specific time after infection if the moderated *t*-test between the infected and pre-infection samples resulted in a *p*-value lower than 0.05 (after Benjamini-Hochberg correction), with an absolute fold change in expression greater or equal to 3, and an average number of reads across all samples greater than 4 counts per million (CPM). When calculating relative changes to the pre-infection samples, infected samples were not subtracted from their individual uninfected controls (since not every infected sample had a pre-infected control), but from an average of all the pre-infection samples. Statistical significance, however, was calculated using the individual pre-infection samples, not their average.

### Fold change analysis of microarray data

Agilent two-color human gene expression microarrays were processed using limma [30]. Fold changes were obtained by calculating the log-ratio between the intensities of the red channel (corresponding to experimental samples) and the green channel (corresponding to Human Universal Reference RNA [31]). These were later background-corrected and normalized using the LOESS algorithm. Agilent one-color mouse gene expression microarrays were processed in a similar manner as the two-color arrays, but using the reported expression value for each probe instead of the log-ratio between the two channels. Benjamini-Hochberg was used to correct the false discovery rate of multiple testing.

## Results

To examine the response of the circulating immune system of nonhuman primates infected with Ebola virus, we used sequencing data from PBMCs of cynomolgus macaques infected with Ebola virus via intramuscular injection. These included pre-infection samples and samples taken 4 and 7 days after infection, as described in [17, 18] (unless specified otherwise, results and figures discussed in this manuscript refer to this dataset). We were first interested in connecting the transcriptomic data to previously reported information about the host cytokine response to Ebola virus infection and to examine whether other cytokines showed evidence of being upregulated during infection.

### Cytokine responses during Ebola virus infection

**Fig. 1:**
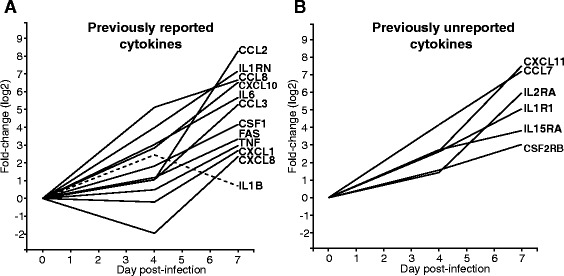
Changes in expression of cytokines during Ebola virus infection. Lines represents the average fold-change in expression (log2, *y-axis*) of each cytokine gene at different times (*x-axis*) after Ebola virus infection. Panel **a** shows twelve cytokines that undergo strong transcriptional changes and that were previously reported to experience changes in serum concentration during Ebola virus infection. IL1B (dashed stroke) shows a transient level of expression, peaking at 4 dpi. Panel **b** shows six cytokines that follow similar patterns of expressions to those in Panel (**a**) but that have not been previously reported to play a role during Ebola virus infection

Previous studies have shown strong increases in cytokine protein levels in the serum of infected animals and humans [32–34]. In our analysis, we observe that a subset of these cytokines reported to be present in the serum also show transcriptional upregulations in PBMCs by 4 days post-infection (IL6, IL1B, IL1RN, CCL8, CXCL10) and by 7 days post-infection (CCL2, CCL3, CSF1, TNF, CXCL1, FAS and CXCL8). Figure 1a shows the average fold-change (log2) for each of these cytokines on the y-axis, as well as the time after infection when the samples were taken on the x-axis. These results suggest a biphasic immune response to infection. IL1B is the only cytokine with an unusual transcription pattern, peaking at day 4 and returning to pre-infection levels by day 7.

Additionally, we identified several cytokines that were not previously known to play a role during Ebola virus infection. Among these, some became upregulated during the early stage of infection (day 4) CCL7, CXCL11, IL1R1 (cytokine receptor) and IL15RA, and others became upregulated during the late stage of infection (day 7): IL2RA, CSF2RB and others (Fig. 1b).

**Fig. 2:**
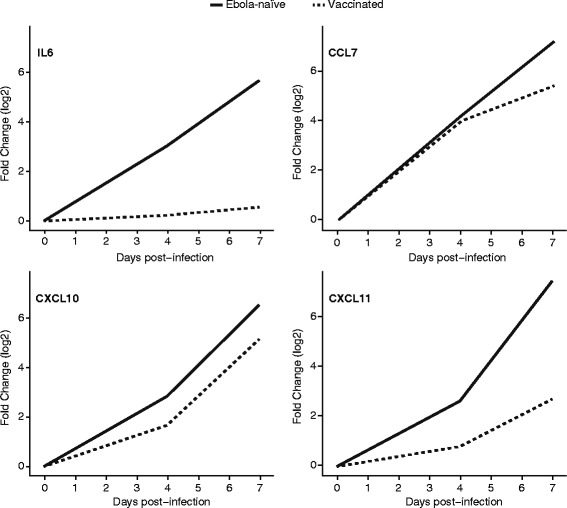
Effects of vaccination in the expression of cytokines after Ebola virus infection. Each line represents the log2 fold-change (*y-axis*) of three cytokines that become upregulated in vaccinated animals: CCL7, CXCL10, and CXCL11 at different times (*x-axis*) after Ebola virus infection. IL6 is shown to illustrate the behavior of a cytokine that only becomes expressed in Ebola-naïve animals

The majority of cytokines during Ebola virus infection in vaccinated animals did not reach a 3-fold change in expression at 4 or 7 days post-infection. Three notable exceptions were CCL7, CXCL10 and CXCL11, which peaked (respectively) at 42.8, 35.5 and 6.6 times the level of expression at 7 days post-infection compared to the pre-infection levels. These genes mirrored the expression patterns seen in the Ebola-naïve animals, but at a lower magnitude (Fig. 2). For example, by 4 days post-infection the expression level of CXCL10 had gone up 7.1-fold in the Ebola-naïve group, but only 3.1-fold in the vaccinated group. By 7 dpi, these values were 92.6-fold in the Ebola-naïve group and 35.5-fold in the vaccinated group.

### A strong and sustained early host transcriptional response after Ebola virus infection contains many genes associated with the innate immune response

**Fig. 3:**
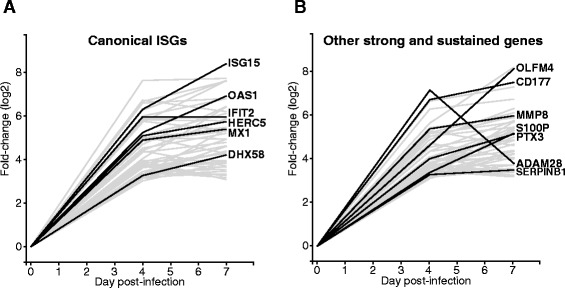
Host genes showing strong and sustained changes in expression early after Ebola virus infection. Each line represents the average fold-change in expression (log2, *y-axis*) of each gene at different times (*x-axis*) after Ebola virus infection. Panel **a** shows canonical interferon-stimulated genes while panel **b** shows other strong and sustained genes. A representative sample of genes is shown in black to illustrate the individual patterns of expression

We also analyzed the macaque intramuscular Ebola dataset to identify genes that underwent strong, statistically-significant increases in expression between pre-infection and 4 days post-infection. The thresholds that we used were 8 or higher fold-change with at least 4 read counts per million, and an adjusted *p*-value of less than 0.05. We found 125 genes that met these requirements, 25 of which showed levels of expression that peaked at 4 days post-infection and decreased by 7 dpi. The levels of expression of the other 100 genes remained constant or increased between days 4 and 7. Of these, 33 genes are known to be responsive to innate immunity transcription factors such as IRF3, IRF7 and STAT1 (also known as interferon-stimulated genes, or ISGs) [35, 36] (Fig. 3a) and are herein referred to as canonical ISGs. Figure 3a shows the log2-fold change of these interferon-stimulated genes at 0, 4 and 7 days post-infection, highlighting a few representative examples (MX1, ISG15, OAS1, DHX58 (RIG-I), IFIT2, and HERC5). The other 47 strong and sustained genes show similar responses to the ISG group but they have not been previously identified as downstream effectors of the interferon response (Fig. 3b). This population includes several neutrophil-associated genes (OLFM4, CD177, SERPINB1, S100P, PTX3 and MMP8), suggesting that there is an accumulation of neutrophils in PBCMs during the course of infection.

**Fig. 4:**
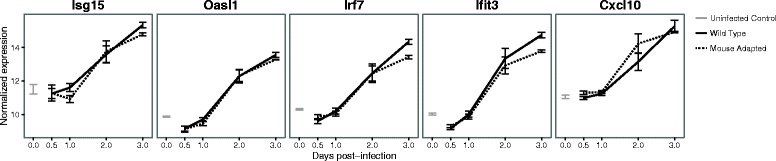
Expression of interferon stimulated genes in spleens of Ebola virus infected mice. Each line represents the average fold-change in expression (log2, *y-axis*) of each gene at different times (*x-axis*) after Ebola virus infection, for each of the conditions: uninfected control, non-pathogenic wild-type Ebola virus infection, and pathogenic mouse-adapted Ebola virus infection

The strong increase in canonical ISGs following Ebola virus infection led us to investigate if this strong interferon-like response was observed in other animal models of Ebola virus infection. To do this, we used the Ebola mouse model dataset to analyze the ISG response in mice that had been infected with wild type EBOV (which is non-pathogenic in mice) and mouse-adapted EBOV (which is pathogenic in mice) [9]. Interestingly, we found that a strong ISG response was apparent 3 days after infection in mice that were infected, irrespective of pathogenesis (Fig. 4).

We validated these results using expression data from two previous studies that infected rhesus macaques with Ebola virus (intramuscular injection) and treated them with an anticoagulant drug (recombinant human activated protein C (rhAPC), and recombinant nematode anticoagulant protein c2 (rNAPc2) [37, 38]). We found significant similarity for a majority of early response genes even though the expression data was quantified using microarrays. For example, in the rhAPC samples, the expression of IFIT2 increased 4 log2-fold and 7.4 log2-fold at 3 and 6 days post-infection, respectively; in the rNAPc2 samples the increases were 4.6 log2-fold and 6.9 log2-fold at 3 and 6 days post-infection, respectively. Additionally, we were also able to confirm these patterns in a previous study that quantified PBMC samples from Ebola virus-infected macaques using microarrays [12]. In this dataset, for example, IFIT2 expression increases 3.4 log2-fold 3 dpi and remains sustained throughout infection. The fact that this multiple transcriptomic studies agree on this observation provides additional evidence that the innate immune signaling is upregulated after infection.

### The sustained transcription of early responsive genes is not present in a non-productive infection

**Fig. 5:**
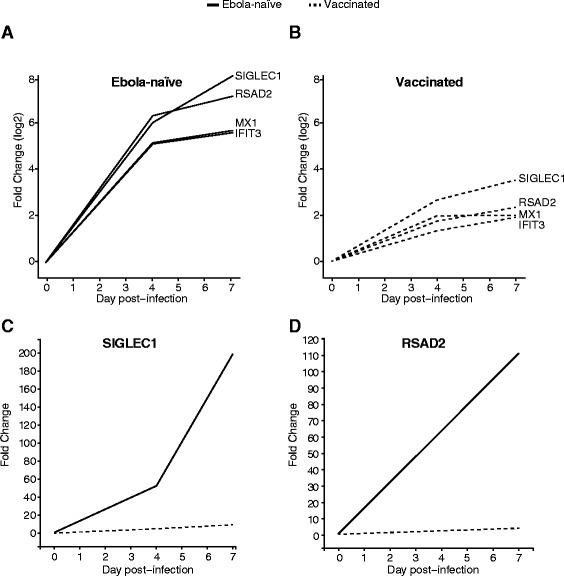
Ebola-naïve and vaccinated responses to Ebola virus infection. Each line represents the average fold-change in expression (log2, *y-axis*) of MX1, RSAD2, SIGLEC1, and IFIT3 at different times (*x-axis*) after Ebola virus infection. Panel **a** shows genes from Ebola-naïve macaques, and Panel **b** shows genes from vaccinated animals. Panels **c** and **d** show the fold-change of SIGLEC1 and RSAD2 in vaccinated and Ebola-naïve macaques

Given the repeated appearance of a type I interferon-like signature following Ebola virus infection, we were interested in determining whether the appearance of an early innate immune response following a hemorrhagic fever virus infection was a response to the injection of a negative strand RNA virus. To do this, we used the intramuscular Ebola dataset to compare the innate immune response to Ebola virus in non-human primates that had been previously vaccinated against Ebola virus to those that had been immunized against a different hemorrhagic fever virus and were therefore susceptible to Ebola virus (Ebola-naïve group) [17, 18]. Figure 5 shows the changes in expression of four ISGs that illustrate the overall differences in response between the two animal cohorts. In the Ebola-naïve group, for example, SIGLEC1 undergoes a 5.72 log2-fold change 4 days post-infection, while in the vaccinated group the change is only 2.53 log2-fold change (Fig. 5a). By 7 days post-infection the difference is even greater: 7.64 in the Ebola-naïve group, and 3.36 in the vaccinated group. A similar trend was seen for RSAD2, MX1, IFIT3, and the majority of genes making up the early transcriptional response: vaccinated animals showed significantly lower levels of expression than Ebola-naïve animals. These results argue that the strong interferon-like response is the result of active virus dissemination, and is not a non-specific response to the injection of viral material.

### The early transcriptional response is common to multiple hemorrhagic fevers

**Fig. 6:**
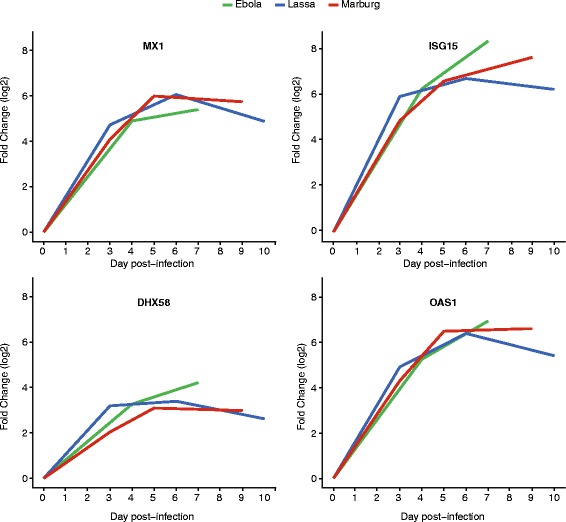
Changes in expression of interferon-stimulated genes during different hemorrhagic fever infections. Each line represents the average fold-change in expression (log2, *y-axis*) of MX1, ISG15, DHX58 or OAS1 at different times (*x-axis*) after Ebola, Lassa or Marburg virus infection

The early host transcriptional response that we observed in these studies appeared to be similar to strong innate transcriptional responses observed in other hemorrhagic fevers [24]. To compare these responses, we used gene expression data from cynomolgus macaques exposed via aerosol to either Lassa virus [24, 39] or Marburg virus [24, 40], and found that the innate response to both infections is highly similar to that of Ebola virus infection. This is illustrated in Fig. 6, which shows the changes in expression of four canonical ISGs (MX1, ISG15, DHX58 and OAS1) during Ebola, Lassa, and Marburg virus infection. All four genes are significantly upregulated at the earliest infected timepoint, and they remain sustained throughout the late stage of disease. For example, MX1 goes up 4.7 log2-fold 3 days after Lassa infection, 4.1 log2-fold 3 days after Marburg infection, and 4.9 log2-fold 4 days after Ebola virus infection. By 6–7 days post-infection, the log2-fold changes are 6.1, 6, and 5.4, respectively. By 9–10 days post-infection, the expression of MX1 seems to decrease mildly in Lassa and Marburg infection, but it remains at very high levels compared to the pre-infection baseline. During Lassa infection, ISG15 and OAS1 undergo similar expression changes to those of MX1: a strong increase, followed by a slight decrease in expression. For Marburg and Ebola virus infection, the early levels of expression of these genes increase more rapidly than during Lassa infection, and this trend continues during the late stage of disease. These results support the hypothesis that the immune system responds to different viral infections via a common, early, sustained and strong innate immune response.

### Different routes of infection lead to a similar early transcriptional response

**Fig. 7:**
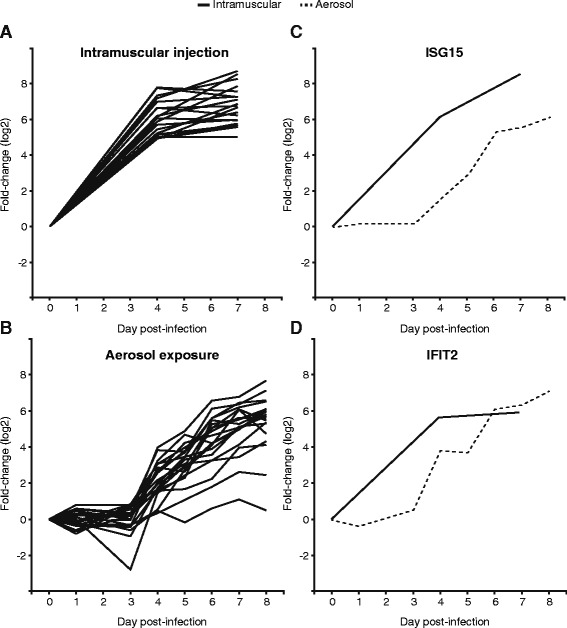
Aerosol infection shows a delayed transcriptional response. Each line represents the average fold-change in expression (log2, *y-axis*) of one of 20 top genes at different times (*x-axis*) after Ebola virus infection. Panel **a** corresponds to intramuscular injection (quantified using RNA sequencing), and Panel **b** to aerosol exposure (quantified using microarrays). Panels **c** and **d** illustrate the delay observed in two individual genes (ISG15 and IFIT2) in the aerosol and intramuscular groups

To determine if the route of infection could alter the early transcriptional response to Ebola virus infection, we looked at gene expression data from a study that exposed macaques to Ebola virus via aerosol and we compared it to the intramuscular injection data. Figure 7 shows the expression of the top 20 genes (Additional file 1) that have the strongest changes 4 days post-infection in the intramuscular group (Fig. 7a), as well as the pattern of expression of those same genes under aerosol exposure (Fig. 7b). RNA sequencing was used to quantify the expression in the intramuscular group, while microarrays were used for the aerosol group. We find that both groups show a strong increase in expression starting at 3–4 days post-infection and increasing during the course of disease. The average log2 fold-change in the intramuscular group at 4 days post-infection is 6.05, and in the aerosol group at 3 days post-infection it is 2.17. Given that this difference in magnitude takes place at different days, it is not possible to determine if the aerosol model shows a delayed response without obtaining additional samples at these timepoints. By 6 days post-infection, the fold change in expression for both models is similar.

### The innate immune response takes place across most tissues

**Fig. 8:**
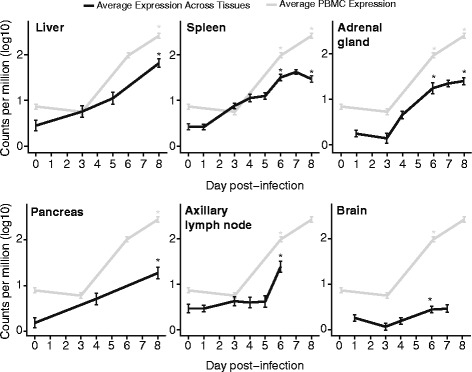
Innate transcriptional response in tissues in an aerosol macaque model of infection. Black lines represent the average expression across all tissues for the 111 genes most highly expressed in PBMCs (*grey line*). The y-axis represents log_10_ read counts per million, and the x-axis different times after infection. Statistically significant differences at matching time points are highlighted with (*)

We next used the list of genes that were strongly upregulated in PBMCs in the macaque intramuscular Ebola dataset (125 genes, Additional file 1) and looked at their expression in the macaque aerosol Ebola dataset. We found that 88 % of the genes from the intramuscular list showed strong upregulation in the aerosol model (111 genes, Additional file 2). We also looked at the expression of these genes in the remaining tissues in the aerosol model and found that the early immune response is present across most tissues at varying magnitudes and expression rates. Figure 8 shows the average expression rate of these genes across each tissue type. PBMCs become transcriptionally activated at 6 days post-infection, and show the strongest activation compared to the remaining tissues at 7 days post-infection. Liver starts becoming transcriptionally activated 3 days post-infection and the average expression level continues to increase by 5 and 8 days post–infection. The gene expression response in the spleen begins to increase at day 3 and also increases to similar levels as in the liver. In the adrenal gland and the pancreas, the expression level increases at 4 days post-infection and remains activated until the end of the infection. The axillary lymph node is the last to become activated, starting around day 6. The brain shows the lowest levels of activation, with only a slight increase that begins on days 4–6.

## Discussion

Our analysis of the transcriptomic response to Ebola virus infection highlights that there is a strong activation of innate immune response genes at early times post-infection, most of which are classified as interferon-stimulated genes. This finding is consistent with other analyses of host responses to different viral infections [14, 24, 41] and with an earlier report [12]. These findings highlight a strong contrast between in vitro studies of Ebola virus function. Previous studies have shown that Ebola virus infection in vitro blocks the expression of ISGs in liver cells [7, 8]. This interferon-antagonism is based on the ability of VP35 to block the activation of IRF3, which inhibits the expression of interferon beta and other ISGs. Mutation of VP35 in a manner that allows IRF3 signaling has been studied in mice [10] and guinea pigs [11] using a recombinant Ebola strain containing a single-point mutation in VP35 (R322A). This mutation reduced the virus’s ability to replicate and to block interferon signaling in vitro; in vivo, this virus did not cause pathogenesis in these animal models. These studies are consistent with the hypothesis that Ebola virus causes a systemic inhibition of the interferon response [42].

A caveat of these earlier studies is that they did not measure the innate immune response in the animals during Ebola virus infection to determine if there was an observable change in interferon-induced signaling in the animal model as well. Our analysis found that mice infected with both pathogenic and non-pathogenic EBOV show a strong expression of interferon-stimulated genes in the spleen. The fact that strong interferon responses occur in mice infected with non-pathogenic WT-EBOV is perhaps not surprising, as the non-adapted virus would be expected to induce a strong innate immune response. That the same response was seen in pathogenic MA-EBOV infected mice argues that a robust innate immune response in the spleen is generated in response to both pathogenic and non-pathogenic EBOV infection in mice. It was reported that in the mouse model the WT-EBOV showed strongly attenuated and delayed growth, while the MA-EBOV showed strong growth [9], perhaps suggesting that the non-mouse adapted virus was suppressed by innate signaling while the pathogenic virus was not.

**Fig. 9:**
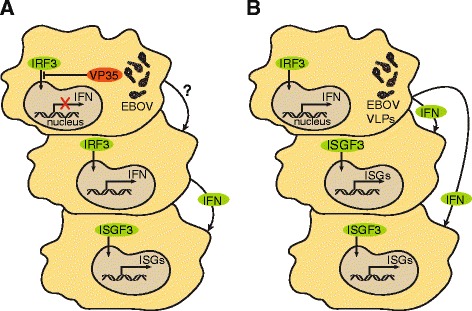
Model for the expression of interferon-stimulated genes during Ebola virus infection. **a** Ebola-infected cell (*top*) is not able to produce interferon due to the VP35-inhibition of IRF3 translocation. We suggest that the infected cell, through an unknown mechanism, might be able to induce neighboring cells (*middle*) to translocate IRF3 to the nucleus and start producing interferon. Once interferon is released by the neighboring cells, it activates the receptors of additional cells (*bottom*) and leads to the transcription of ISGs. **b** An alternative model is that some cells (*top*) can become infected with Ebola VLPs, which are not able to block IRF3 translocation, and therefore they can produce interferon, release it to neighboring cells (*middle* and *bottom*) and they in turn start transcribing ISGs

This observation that innate immune genes are upregulated in the mouse-model of EVD is consistent with earlier reports suggesting that primary cells exposed to Ebola virus strongly activate interferon-like signaling [43] and data showing that in NHP-infection interferon-like signaling is upregulated [12, 38]. Together, the findings from two different model systems imply that innate signaling is largely unimpeded during Ebola virus infection and would be observed in human disease as well. Either IRF3 is activated or interferon is expressed and released into the circulation leading to an early, and global innate response in vivo. Additional experiments are needed to determine why we detect a significant increase in the expression of interferon-stimulated genes at the early stages of Ebola virus infection. One explanation could be that infected cells stimulate uninfected neighboring cells to produce interferon through a currently unknown mechanism, and that this stimulates them to express ISGs and to continue spreading the interferon signal (Fig. 9a). Since we see viral titers and ISG expression increasing throughout infection, further studies are required to understand if the populations of cells that are expressing ISGs are different from the ones that are undergoing viral replication. Another hypothesis is that, in vivo, some cells may retain their ability to translocate IRF3 to the nucleus and to express interferon, perhaps due to a missing VP35 (Fig. 9b). This is consistent with earlier reports showing that there are early immune-associated transcriptional responses in primary target cells exposed to either Ebola virions or virus-like particles (VLPs) [44, 45].

One of the hallmarks of Ebola virus infection is dysregulated levels of circulating proinflammatory cytokines. We observed the levels of expression of previously reported cytokines such as IL6, and CXCL8 (IL8) increase between 4 and 7 days post-infection. We also found several cytokines, such as CCL7 and CXCL11 (I-TAC), that showed significant changes in expression but whose protein levels have not been previously observed during Ebola pathogenesis. CXCL10 (IP-10) and CXCL11 were two of the cytokines that were expressed in animals that succumb to infection. Interestingly, we saw a significant increase in expression in vaccinated macaques infected with Ebola virus. This suggests that they are part of a conserved response in both the pathogenic and non-pathogenic response to Ebola virus. Both cytokines are induced by interferon alpha (they are ISGs), share a common receptor (CXCR3), and are thought to be involved in the recruitment of effector T cells and NK cells [46]. They are also known to be induced during the acute phase in other types of viral infections including dengue [47], influenza [48], hepatitis B and C [49, 50], Herpes simplex [51] and HIV-1 [52]. Upregulation of CXCL10 has also been associated with hemorrhagic manifestations in patients infected with Sudan virus [34]. Our observations suggest that these cytokines are part of a core innate immune response that is triggered in all animals exposed to Ebola virus, but that they are overexpressed in animals that will succumb to disease.

By comparing how different routes of infection affect the cellular circulating immune response in Ebola virus-infected primates, we observed that infection via an aerosol and via intramuscular injection resulted in similar patterns of gene expression. Previous studies have reported a delay in the aerosol model of exposure compared to the intramuscular model [53], but given that we do not have samples from both infections taken at identical early time points, we were not able to confirm this observation.

## Conclusions

Infection with Ebola virus leads to early and robust interferon-like responses that take place before the appearance of virus in the blood. This response takes place not only on circulating immune cells, but throughout the majority of infected tissues. Our results extend earlier observations of a strong innate immune response and suggest the involvement of new cytokines in Ebola virus infection. Further analysis of the cells responsible for driving this response, and for producing the different cytokine signals, will be important to understand the ability of the virus to replicate virtually unchecked in many tissues—even when these tissues show a strong interferon-expression signal—, and to identify which cells are undergoing an uncontrolled ISG response.

## Additional files

Additional file 1: 125 strongly upregulated genes in PBMCs in the macaque intramuscular Ebola dataset. For each gene, we report the log fold-changes at 4 and 7 days post-infection, the corresponding adjusted *p*-values and its functional category. (XLSX 20 kb) Additional file 2: 111 strongly upregulated genes in PBMCs in the macaque aerosol Ebola dataset. For each gene, we report the log fold-change at 6 days post-infection, the corresponding adjusted *p*-values and its functional category. (XLSX 14 kb)

## Abbreviations

CPM: Counts per million
EBOV: Ebola virus
GEO: Gene expression omnibus
ISG: Interferon stimulated gene
LASV: Lassa virus
MARV: Marburg virus
PBMC: Peripheral blood mononuclear cells
PFU: Plaque-forming units
rhAPC: Recombinant human activated protein C
rNAPc2: Recombinant nematode anticoagulant protein c2
rVSV: Recombinant vesicular stomatitis virus with the ebola glycoprotein
VLP: Virus-like particle
